# FIRST POSTPARTUM HOME VISIT: A PROTECTIVE STRATEGY FOR EXCLUSIVE BREASTFEEDING

**DOI:** 10.1590/1984-0462/;2018;36;1;00001

**Published:** 2018-01-15

**Authors:** Maria José Laurentina do Nascimento Carvalho, Michelle Figueiredo Carvalho, Carlos Renato dos Santos, Paula Thianara de Freitas Santos

**Affiliations:** a Universidade Federal de Pernambuco, Vitória de Santo Antão, PE, Brasil.

**Keywords:** Breastfeeding, Home visit, Weaning, Aleitamento materno, Visita domiciliar, Desmame precoce

## Abstract

**Objective:**

To investigate the influence of the first postpartum visit, family income, pacifier habit, number of siblings and birth weight on the maintenance of exclusive breastfeeding in infants aged one week up to six months.

**Methods:**

In this cross-sectional study, data were collected through a survey, which included social and demographic characteristics of the families and the breastfeeding practice in children aged one week to six months, who received care at family health units in the municipality of Vitória de Santo Antão, Pernambuco, Northeast Brazil, between December 2014 and February 2015. Prevalence ratio was used to indicate how many times the outcome prevalence was increased by the influence of the studied variables, as well as a binary logistic regression model for the analysis and reliability of the results.

**Results:**

The prevalence of exclusive breastfeeding was 41.7%. Family income, pacifier habit, number of siblings and birth weight did not show statistical association with the maintenance of exclusive breastfeeding. However, the absence of postpartum home visits adversely influenced the outcome (*p*=0.009). The children who received home visits had a higher chance of being exclusively breastfed for six months or more (PR 2.28, 95%CI 1.17-4.42). In the logistic regression, only the visit showed significance to estimate the probability of exclusive breastfeeding.

**Conclusions:**

The absence of postpartum home visits negatively influenced the duration of exclusive breastfeeding. This finding fills a gap in the knowledge of determinants of exclusive breastfeeding and may guide the planning of local strategies and actions to promote, protect and support exclusive breastfeeding.

## RESUMO

**Objetivo:**

Averiguar a influência da primeira visita puerperal, da renda familiar, do hábito de chupeta, do número de irmãos e do peso ao nascer na manutenção do aleitamento materno exclusivo (AME) em lactentes com uma semana de vida até seis meses de idade no município de Vitória de Santo Antão, Pernambuco.

**Métodos:**

Neste estudo transversal, coletaram-se dados por inquérito que abrangiam características sociais e demográficas das famílias e prática de amamentação em crianças com uma semana até seis meses de vida, que compareceram às unidades de saúde da família de Vitória de Santo Antão nos dias de puericultura, entre dezembro de 2014 e fevereiro de 2015. Para indicar quantas vezes a prevalência do desfecho foi aumentada por influência dos fatores analisados, utilizou-se a Razão de Prevalência, bem como um modelo de regressão logística binária para análise e confiabilidade dos resultados.

**Resultados:**

A prevalência de amamentação exclusiva foi de 41,7%. A renda familiar, o hábito de chupeta, o número de irmãos e o peso ao nascer não demonstraram significância estatística sobre a manutenção do AME. Em contrapartida, a ausência da visita puerperal (*p*=0,009) influenciou negativamente a sua permanência. As crianças que receberam visita mostraram maior possibilidade de estarem em AME (RP 2,28, IC95% 1,17-4,42). Na regressão logística apenas a visita apontou significância para estimar a probabilidade de ocorrer AME.

**Conclusões:**

A ausência da visita puerperal influenciou negativamente a manutenção do AME. Esse achado preenche a lacuna referente ao conhecimento dos fatores determinantes sobre essa prática e norteia o planejamento de ações e estratégias locais para promoção, proteção e apoio à amamentação exclusiva.

## INTRODUCTION

It is agreed that breastmilk is the best food for the promotion and protection of children’s health, and has recognized nutritional, immunological, cognitive, economic, social and emotional benefits.^1^ ^,^ ^2^ These benefits are exploited to the fullest when breastfeeding is practiced exclusively up to six months of age, being supplemented by other types of food up to two years of age or more, as recommended by the World Health Organization (WHO).^3^

The WHO estimations have shown that if breastfeeding were practiced universally, the deaths of 823,000 children and 20,000 mothers could be prevented each year.^4^ In spite of the benefits of exclusive breastfeeding and of the efforts to promote it, rates in Brazil, although with an evident improvement,^5^ are below the recommended levels, and its early interruption is an important public health problem.^6^ The most recent survey on the prevalence of BF in Brazilian capitals and in the Federal District, published in 2009, revealed a prevalence of 41% of BF. Even though there was a significant increase in the indexes on the fourth month of life in Brazil (from 35.5% in 1999 to 51.2% in 2008), this rate does not reach 10% in the sixth month.^7^

The Family Health Strategy is a privileged space for actions to promote, protect and support BF. In this context, it is possible to develop educational practices for the promotion of BF from prenatal care, which should be part of the health team’s entire agenda, and is essential to establish a connection between this and the mother-child binomial and families, providing support, clarification on the common intercurrences in breastfeeding and health promotion.^8^ The National Strategy for the Promotion of Breastfeeding and Healthy Complementary Food in the Unified Health System (SUS) - The Brazilian Breastfeeding Strategy (EAAB), launched in 2012, aims to qualify the actions in this scenario through the improvement of the competences and skills of health professionals in Basic Health Units (BHU). This strategy adds important efforts to the Family Health Strategy regarding BF, qualifying the work process through critical-reflexive lifelong education and contributing more effectively to the increase in BF rates.^9^

The maintenance of exclusive BF can be influenced by several factors. The postpartum home visit, one of the activities inherent to the Family Health Strategy, is a fundamental intervention instrument in family health, as it enables the professional to have more contact with the mother-child-family trinomial, bringing them closer to the reality experienced and the main health needs.^10^ This is recommended in the first week after the infant is discharged from the hospital, and in the first three days if the newborn has been classified as being at risk. The main objectives of the visit are to evaluate the health status of the mother and the newborn and the interaction between them; to guide and support the family on breastfeeding and basic care for the newborn; to guide family planning and to identify risk situations or possible intercurrences for the adoption of appropriate practices.^11^

Other BF determinants should be listed. *Per capita* family income is one of them, as it is believed that adherence to breastfeeding is positive when there is a better level in this aspect,^1^ although there is no consensus among the studies that made this association.^1^ ^,^ ^12^ ^,^ ^13^ The pacifier habit is a predictive factor for the interruption of exclusive BF, since it can reduce the frequency with which the child sucks on the breast, reducing the production of breast milk and changing the infant’s oral dynamics, which reinforces the orientation that the mothers should not offer it to the newborn;^6^ however, studies are still controversial.^6^ ^,^ ^14^ ^,^ ^15^ ^,^ ^16^ The number of children is another relevant aspect, since it is closely related to previous experiences with breastfeeding. There is an increase in the prevalence of exclusive BF among mothers who have breastfed, correlating with their previous experiences.^17^ Another largely studied factor is birth weight, since children with low weight, due to their immature condition, present greater difficulty in sucking and shorter duration of breastfeeding, which may compromise the success of exclusive BF.^6^ ^,^ ^12^ ^,^ ^16^

In view of the above, the present study aimed to determine the influence of the first postpartum home visit, family income, pacifier habit, number of siblings and birth weight in the maintenance of exclusive BF in infants aged from one week to six months in the city of Vitória de Santo Antão, Pernambuco.

## METHOD

This is a cross-sectional quantitative study that presents the results of a broader research entitled “Evaluation of health and nutrition conditions of mothers and infants receiving care at health units in the city of Vitória de Santo Antão, PE, Brazil”, carried out between 2014 and 2015.

The data were collected through a standardized instrument, applied to mothers, with questions formulated from the recommendations of the Prenatal and Birth Humanization Program and the Child Health Care Program. It is a pre-coded questionnaire on socioeconomic, demographic and prenatal care conditions, duration of BF and causes of weaning.

The sample consisted of 62 children, accompanied by their mothers, who were assisted in the Family Health Units (FHU) in the urban and rural areas of the city of Vitória de Santo Antão, Pernambuco, representing 72.6 and 27.4%, respectively, of the interviews conducted between December 2014 and February 2015 in the morning and afternoon periods. The study included 15 FHUs; however, the distribution of infants was not equal between them. All mothers agreed to participate in the research and signed the informed consent form. As inclusion criteria, we considered: children with one week of life, since the puerperal visit happens from the first week postpartum to the sixth month of age; and nursing mothers of the covered areas of the municipality who were assisted in the FHU on the day of collection and presented no complications during pregnancy or after delivery, which contraindicates breastfeeding. The percentage of loss was 3% (n=2) due to the lack of information in the questionnaires.

The main variables explored were: socioeconomic level (<1 and ≥1 minimum wage); number of siblings (1, 2 and ≥3); puerperal home visit performed in the first week postpartum (yes, no) by the Family Health Strategy professional, as recommended by the Ministry of Health (MS);^11^ pacifier use (yes, no); weight at birth (categorized as <2500 g - low weight and ≥2500 g - adequate weight) according to the MS;^11^ aspects related to breastfeeding - infant is being or were breastfed (yes, no), and whether or not other foods were offered during breastfeeding. For the latter, more than one response was considered by the mother in the analysis of the data, that is, the same child could have received one or more of the foods mentioned: water, tea, infant formulas, powdered cow’s milk and juice.

The EpiData software (The EpiData Association, Odense, Denmark) was used for the construction of the database, and the Statistical Package for the Social Sciences (IBM Corp. Armonk, NY, USA) was used for the statistical analysis of the variables. The descriptive analysis was performed with frequency calculations of the results found, and the association between the variables was investigated through the chi-square test, with the outcome variable being exclusive BF and adopting a level of significance lower than 0.05 (p<0.05) for all analyses. To indicate how many times the prevalence of the outcome was increased by factors of influence, the measure of association used was the Prevalence Ratio.

Due to the nature of the variable dichotomous response (presence or not of exclusive BF), a binary logistic regression model was set up, which consists of a particular case of the generalized linear models.^18^ To select the best model, the method proposed by Akaike^19^ was chosen, which differs from other model selection procedures because it is a minimization process that does not involve statistical tests. A logistic regression model using the dependent variable exclusive BF (0 - no and 1 - yes) and independent variables *X*_*1*_: presence of puerperal visit (0 - no and 1 - yes); *X*_*2*_: age of the child (1 - up to two months and 2 - more than two months); *X*_*3*_: income (1 - ≤1 minimum wage and 2 - >1 minimum wage); e *X*_*4*_: sex (1 - male and 2 - female) was adjusted using the *glm* routine of software R (R Core Team - R Foundation for Statistical Computing, Vienna, Austria).

This study was authorized by the Human Research Ethics Committee at Universidade Federal de Pernambuco (UFPE), according to protocol no. 390191 and CAAE no. 15371413.8.0000.5208.

## RESULTS

The characteristics of the infants studied are shown in Table 1. The majority were male and presented adequate birth weight (≥ 2500 g). Most of the infants had a sibling, and the mothers lived with their spouse and children. In relation to basic sanitation, the majority had piped water, the waste disposal was carried out by means of sewage network and the garbage was collected. As for the socioeconomic aspect, the family income concentrated below one minimum wage, and most families reported not receiving government benefits.

**Table 1:** Characteristics of the of children aged one week to six months receiving care in the Family Health Units and sociodemographic, economic and housing parameters of the families in the city of Vitória de Santo Antão, Pernambuco, 2014-2015.

Variables (n=62)	n	%
Sex		
Female	25	40.3
Male	37	59.7
Age*		
1 week to ≤3 months	38	61.3
>3 months to ≤6 months	24	38.7
Birth weight** (g)		
<2500	5	8.1
≥2500	55	88.7
Number of siblings		
One	29	46.8
Two	16	25.8
Three or more	17	27.4
Lives with***		
Spouse and children	41	66.1
Spouse and family	9	14.5
Relatives	9	14.5
Piped water		
Yes	54	87.1
No	8	12.9
Waste disposal		
Cesspool	20	32.3
Open pit	5	8.1
Sewerage system	37	59.7
Garbage disposal		
Garbage collection	54	87.0
Burnt	6	10.0
Open pit	2	3.0
Family income (minimum wage)		
<1	44	71.0
≥1	18	29.0
Receiving any government benefit****		
Yes	29	46.8
No	33	53.2

*Categorization was not considered in the statistical analysis; **Two questionnaires did not contain this information; ***Three questionnaires did not contain this information; ****The most cited benefit was Bolsa Família (32.2%; n=20).

The prevalence of exclusive BF, BF and weaned children was 41.7% (n=25), 38.3% (n=23) and 20.0% (n=12), respectively. Most infants had received some type of food - among which the introduction of water and tea in the first six months can be highlighted - and the most frequent justification given by mothers for having weaned or discontinued exclusive BF was insufficient milk. Most of the children used pacifiers. Detailed information regarding BF is shown in Table 2.

**Table 2:** Characteristics related to breastfeeding. Vitória de Santo Antão, Pernambuco, 2014-2015.

Variables (n=62)	n	%
Prevalence*		
Exclusive breastfeeding	25	41.7
Breastfeeding	23	38.3
No breastfeeding	12	20.0
Justifications for exclusive BF weaning/discontinuation		
Insufficient milk	10	16.1
Infant did not want it	2	3.2
Mother worked or studied	2	3.2
Mother did not want to	1	1.6
Received another type of food while breastfeeding**		
Yes	33	53.2
No	25	40.3
Foods offered in the first six months***		
Water	27	43.5
Tea	16	25.8
Baby formulas	14	22.6
Powdered whole cow’s milk	11	17.7
Juice	10	16.1
Pacifier		
Yes	33	53.2
No	29	46.8

AME: aleitamento materno exclusivo; *Dois questionários não apresentavam tais informações; **Dois questionários não tinham tal informação e em outros dois as crianças nunca tinham recebido leite materno; ***Pode haver mais de uma resposta para a mesma criança.

The data described in Table 3 refer to the characteristics of the sample and the associations obtained between exclusive BF and the variables investigated in this study. Emphasis should be given to the puerperal visit, in which it was evaluated whether or not the child was seen in the first week of life by a health professional of the Family Health Strategy, as recommended by the MS,^11^ observing that 48.4% (n=30) received the visit, and 51.6% (n=32) did not. The most cited visiting professionals: nurse (30.6%, n=19), community health agent (22.6%, n=14), physician and speech pathologist (3.2%, n=2 each), and nutritionist and physiotherapist (both with 1.6%, n=1).

**Table 3:** Influence of selected variables on the maintenance of exclusive breastfeeding in infants aged one week to six months in the city of Vitória de Santo Antão, Pernambuco, 2014-2015.

	Exclusive breastfeeding		p-value
	Yes [n (%)]	No [n (%)]	
Post-partum visit			
Yes	17 (29.3)	11 (18.9)	0.009
No	8 (13.7)	22 (37.9)	
Family income (minimum wage)			
<1	15 (25.8)	25 (43.1)	0.199
≥1	10 (17.2)	8 (13.7)	
Pacifier habit			
Yes	10 (17.2)	19 (32.7)	0.185
No	15 (25.8)	14 (24.1)	
Number of siblings			
One	14 (24.1)	13 (22.4)	0.110
Two	3 (5.1)	12 (20.6)	
Three or more	8 (13.7)	8 (13.7)	

The puerperal visit remained statistically associated with maintenance of exclusive BF (Prevalence Ratio - PR=2.28; 95% confidence interval - 95%CI 1.17-4.42; Table 4). In contrast, family income, pacifier use, and number of siblings showed no significant association.

**Table 4:** Association between the selected variable and exclusive breastfeeding in children aged one week to six months.

	Prevalence ratio	95%CI
Puerperal visit		
Yes	2.2	1.17-4.42
No	1.0	

95%CI: 95% confidence interval.

The logistic regression model showed only the presence of the puerperal visit (*X*_*1*_ ) as being significant to estimate the probability of exclusive BF. The stepwise method and the analysis of significance of the parameters were then used to search for the best model, pointing to a new and reduced one containing only exclusive BF as a dependent variable and the presence of the puerperal visit as an independent variable. A probability of 0.266 of exclusive BF was found to occur in the absence of the visit, and 0.607 in the presence of the visit. The two models presented here demonstrated Akaike’s information criterion of 79.3 and 76.3, respectively. Thus, it is evident that the puerperal home visit is an important factor for the increase in the probability of exclusive BF.

## DISCUSSION

The scientific evidence supporting the excellence of exclusive BF is endless.^1^ ^,^ ^2^ ^,^ ^3^ ^,^ ^4^ ^,^ ^5^ ^,^ ^6^ ^,^ ^20^ However, in Vitória de Santo Antão, Pernambuco, there are few population studies that seek to evaluate maternal and child health conditions and, mainly, to understand the situation of BF and its determinants, underlining the relevance of the present study. In addition, the study is the first to address the puerperal visit and its possible influence on the maintenance of exclusive BF, a fundamental aspect of the Family Health Strategy’s support for the mother-infant binomial and family members.

Even with the recommendations for breast milk to be offered until up to six months of age,^3^ the indicator was lower than expected in the infants evaluated, showing a prevalence of exclusive BF of 41.7%. This low prevalence in this study was similar to that found in other studies with children aged less than six months.^6^ ^,^ ^21^ The reasons for mothers to interrupt exclusive BF, such as insufficient milk, refusal of the child, maternal work or study commitments, corroborate those reported by other authors.^21^ ^,^ ^22^

The expectation was to obtain more satisfactory indicators, since the municipality presents services related to the mother-child network such as the Center for Women’s Health, Policlínica da Criança, the Association for Protection of Childhood Maternity in Vitória and Hospital João Murilo de Oliveira.^23^ The municipality also has the Family Health Strategy and the Community Health Agents Program, with an estimated coverage of 77.02 and 90.30%, respectively, in addition to the Family Health Support Center (FHSC).^24^ These strategies add important efforts to the promotion, protection and support of breastfeeding.^25^ Even though the proportion of children in exclusive BF was below the international recommendations,^3^ ^,^ ^4^ the region, judging by the structure of the network, demonstrates involvement and commitment with the mother-child binomial, and the present study provides subsidies to direct action planning from the understanding of the factors that permeate the practice of exclusive BF.

Several reasons may influence the permanence of exclusive BF, and its understanding is crucial to allow advances in the situation of breastfeeding. Thus, during the first six months of the infant’s life, among the variables tested, the puerperal visit was one that had most influence on the maintenance of exclusive BF. In relation to this aspect, the visit is understood as a privileged moment for the extension of care, important for the mothers to clarify their doubts and for the professional to check the technique and difficulties related to breastfeeding. In addition, the first days after delivery correspond to a critical stage for initiation and maintenance of BF, since it represents a moment of insecurity and emotional fragility for women. As a result, this monitoring provides the development of maternal and family safety and a safe exclusive BF practice.^26^ In the present study, infants who received the puerperal visit showed higher chances to be in exclusive BF.

Based on the above, it can be inferred that the puerperal visit acts as a protective factor in the maintenance of exclusive breastfeeding. It is an opportune moment to carry out healthcare and health education activities that will have a positive impact on maternal and infant health. The support of the professional, through qualified listening, humanized attention, clarification of doubts, guidance of the correct breastfeeding technique and support in coping with possible difficulties in the process contributes with women initiating and maintaining exclusive BF in a more effective manner, collaborating to increase its indexes. In this study, only 48.4% of the mother-infant binomials received visits, most of them performed by the nursing professional and community health agent, revealing a gap in the conception of work based on a multiprofessional logic. The need for greater involvement of physicians and FHSC professionals is emphasized here, since few visits were made by them. It is necessary that this practice becomes a role for everyone to ensure greater effectiveness in the actions and extension in the duration of exclusive BF. It is important to note that, regarding the visit by the community health agent, Oliveira *et al* ^12^ did not identify the influence of this variable on the duration of BF.

Family income was not associated with exclusive BF, despite the hypothesis that the lower income is related with its early interruption, a premise that can be confirmed in the study by Barbosa *et al*.^13^ For these authors, family income lower than or equal to three minimum wages is increased by three times the chance of early weaning. It is believed that these are individuals in an unfavorable socioeconomic situation, with a high number of residents per household, justifying the need for the mother to work to contribute with the family’s subsistence and making it difficult to establish exclusive BF. In the present study, sample size possibly contributed to the lack of such association.

The pacifier habit was present in 53.2% of the sample, but its influence on BF exclusivity was not verified, a result also evidenced by Pellegrinelli *et al.* ^27^ However, there is no consensus on the adverse effects of pacifiers and many results are controversial in the literature.^6^ ^,^ ^14^ ^,^ ^15^ ^,^ ^16^Boccolini *et al* ^16^ pointed out that the use of pacifiers was the aspect most strongly associated with the early interruption of exclusive BF in the studies evaluated. Its use may imply a decrease in the frequency of sucking, resulting in less stimulation of the nipple-areolar complex and consequent reduction in the production of breast milk. There is a clash in the literature on the issue of the pacifier not being the primary reason for weaning, but rather an indication of the maternal willingness to wean or a proof of difficulties with the practice of breastfeeding.^16^ ^,^ ^28^ Sample size may have been a compromising factor in the current study, making it difficult to elucidate the controversy regarding pacifier use.

In relation to the number of siblings, no association with the interruption of exclusive BF was observed. This finding differs from those in other studies,^14^ ^,^ ^16^ ^,^ ^29^ ^,^ ^30^ in which multiparous women, because of previous experiences, offered breast milk exclusively more than primiparous women, implying an increase in the prevalence of exclusive BF. It is worth noting that this variable was not controlled, because it was impossible to identify whether the siblings reported were children with consanguineous ties with the women or not, and that could have influenced the result.

Birth weight is indicated in the literature as a predictive factor for the interruption of exclusive BF, as children with low weight (<2500 g) present more difficulties in the establishment of BF due to immaturity in the frequency and suction pressure, in addition to long periods in a neonatal unit, prolonging the separation from their mothers.^12^ ^,^ ^14^ ^,^ ^16^ In this study, birth weight was not associated with exclusive BF, as verified by other authors.^6^ ^,^ ^14^ However, such a finding may have occurred because of the small proportion of underweight children, as the distribution in the categories did not occur uniformly.

It is worth mentioning that the supply of water, teas, juices and milk from other origins, something common in the population and strongly rooted in the culture, was observed in the present study. This practice is considered harmless by mothers and family members, being encouraged by more experienced family figures such as grandmothers,^28^ and expecting to cure problems such as cramps and gas and to quench the baby’s thirst.^12^ ^,^ ^22^ In this study, water was the liquid most offered by mothers, followed by tea, which is consistent with findings by Campos *et al*.^22^ The introduction of liquids and foods at this age causes a decrease in breast milk intake and a reduction in breast milk production, and it also affects the duration of BF, decreases the mother’s confidence and safety, and exposes the infant to contamination risks.^28^

It is worth mentioning that one of the limitations of the study refers to the results translating the specific reality of a group of children aged up to six months old, highlighting the need for more comprehensive studies to infer the results for the population. Another limitation is related to the non-inclusion of maternal characteristics (age, schooling, profession, type of delivery) for the analysis of the association with exclusive BF, since the collection instrument prioritized questions about the infant.

Finally, the present study pointed out that the prevalence of exclusive BF was lower than that recommended by the WHO, and the aspect that negatively influenced its maintenance was the absence of the puerperal visit. In contrast, family income, number of siblings, birth weight, and pacifier habit showed no significant association. The findings of the current study are of great relevance, since they fill a gap in the knowledge of the determining factors on the practice of exclusive BF in Vitória de Santo Antão, guiding the planning of local actions. Thus, actions in maternal and infant health in the municipality should prioritize the first puerperal visit as a strategy to support and encourage the maintenance of exclusive BF.
